# Youth Depression Alleviation with Anti-inflammatory Agents (YoDA-A): a randomised clinical trial of rosuvastatin and aspirin

**DOI:** 10.1186/s12916-019-1475-6

**Published:** 2020-01-17

**Authors:** Michael Berk, Mohammadreza Mohebbi, Olivia M. Dean, Sue M. Cotton, Andrew M. Chanen, Seetal Dodd, Aswin Ratheesh, G. Paul Amminger, Mark Phelan, Amber Weller, Andrew Mackinnon, Francesco Giorlando, Shelley Baird, Lisa Incerti, Rachel E. Brodie, Natalie O. Ferguson, Simon Rice, Miriam R. Schäfer, Edward Mullen, Sarah Hetrick, Melissa Kerr, Susy M. Harrigan, Amelia L. Quinn, Catherine Mazza, Patrick McGorry, Christopher G. Davey

**Affiliations:** 1 Orygen, the National Centre of Excellence in Youth Mental Health, Melbourne, Australia; 2 Centre for Youth Mental Health, University of Melbourne, Parkville, Australia; 3 The Institute for Mental and Physical Health and Clinical Translation, Deakin University, Geelong, Australia; 4 Florey Institute for Neuroscience and Mental Health, University of Melbourne, Parkville, Australia; 5 Department of Psychiatry, University of Melbourne, Parkville, Australia; 6 Barwon Health, PO Box 281, Geelong, Victoria 3220 Australia; 7 Biostatistics Unit, Faculty of Health, Deakin University, Geelong, Australia; 8 Orygen Youth Health, Northwestern Mental Health, Melbourne, Australia; 9 Black Dog Institute, University of New South Wales, Sydney, Australia; 10 Melbourne School of Population and Global Health, University of Melbourne, Melbourne, Australia; 11 Department of Psychological Medicine, University of Auckland, Auckland, New Zealand; 12 Department of Social Work, Monash University, Melbourne, Australia

**Keywords:** Depression, Treatment, Statins

## Abstract

**Background:**

Inflammation contributes to the pathophysiology of major depressive disorder (MDD), and anti-inflammatory strategies might therefore have therapeutic potential. This trial aimed to determine whether adjunctive aspirin or rosuvastatin, compared with placebo, reduced depressive symptoms in young people (15–25 years).

**Methods:**

YoDA-A, Youth Depression Alleviation with Anti-inflammatory Agents, was a 12-week triple-blind, randomised, controlled trial. Participants were young people (aged 15–25 years) with moderate to severe MDD (MADRS mean at baseline 32.5 ± 6.0; *N* = 130; age 20.2 ± 2.6; 60% female), recruited between June 2013 and June 2017 across six sites in Victoria, Australia. In addition to treatment as usual, participants were randomised to receive aspirin (*n* = 40), rosuvastatin (*n* = 48), or placebo (*n* = 42), with assessments at baseline and weeks 4, 8, 12, and 26. The primary outcome was change in the Montgomery-Åsberg Depression Rating Scale (MADRS) from baseline to week 12.

**Results:**

At the a priori primary endpoint of MADRS differential change from baseline at week 12, there was no significant difference between aspirin and placebo (1.9, 95% CI (− 2.8, 6.6), *p* = 0.433), or rosuvastatin and placebo (− 4.2, 95% CI (− 9.1, 0.6), *p* = 0.089). For rosuvastatin, secondary outcomes on self-rated depression and global impression, quality of life, functioning, and mania were not significantly different from placebo. Aspirin was inferior to placebo on the Quality of Life Enjoyment and Satisfaction Questionnaire (Q-LES-Q-SF) at week 12. Statins were superior to aspirin on the MADRS, the Clinical Global Impressions Severity Scale (CGI-S), and the Negative Problem Orientation Questionnaire scale (NPOQ) at week 12.

**Conclusions:**

The addition of either aspirin or rosuvastatin did not to confer any beneficial effect over and above routine treatment for depression in young people. Exploratory comparisons of secondary outcomes provide limited support for a potential therapeutic role for adjunctive rosuvastatin, but not for aspirin, in youth depression.

**Trial registration:**

Australian New Zealand Clinical Trials Registry, ACTRN12613000112763. Registered on 30/01/2013.

## Background

Depression is the most prevalent and disabling health problem in young people, [1] and its prevalence might be increasing [2]. The peak period for depression onset is youth and early adulthood. It has deleterious social, educational, and developmental effects [3, 4], and can lead to recurrent major illness episodes [5, 6].

It is uncertain if antidepressants are effective in youth depression, with the possible exception of fluoxetine [7, 8]. Notably, a companion study to YoDA-A, Youth Depression Alleviation-Combined Treatment (YoDA-C), which compared fluoxetine to placebo in youth receiving cognitive behaviour therapy, failed to detect a significant main effect of fluoxetine [9]. Also, the monoamine hypothesis [10] has not resulted in truly novel therapies beyond modifications of established agents [11, 12]. Therefore, there is a clear need for the development of effective adjunctive interventions that might be acceptable to young people experiencing depression [13].

Depression is associated with a complex picture of increased immune activation, impaired immune function, and inflammation [14–16], including in young people [17]. Depression not only is associated with depression in youth, but risk factors for depression themselves, such as trauma and obesity, are associated with inflammation [18, 19]. Higher levels of C-reactive protein are associated with risk for the development of de novo depression, suggesting that inflammation contributes at least in part to the genesis and progression of depression [20]. In adolescent depression, inflammation is predictive of therapeutic response, suggesting a core role of these pathways [21].

Statins (3-hydroxy-3-methylglutaryl coenzyme A reductase inhibitors) such as rosuvastatin lower peripheral inflammatory markers in animal [22] and human studies [23]. Aspirin, a cyclooxygenase inhibitor, also reduces systemic inflammatory markers [22, 23]. Statins additionally increase tryptophan levels, a serotonin precursor, by blocking the enzyme indoleamine-pyrrole 2, 3-dioxygenase (IDO) [24]. These mechanisms of action of aspirin and statins overlap with putative pathophysiological pathways in depression, suggesting therapeutic potential [25].

Epidemiological studies suggest that people taking aspirin or statins might be less likely to have concurrent depression [26–30], although the evidence is inconsistent, with some negative reports [31]. While there are positive randomised controlled trials of statins for the treatment of depression [32–34], and meta-analytic evidence for their effectiveness [35], no studies have investigated their therapeutic potential among youth. A pilot study comparing low-dose aspirin added to sertraline with sertraline alone found that the former was superior on the Beck Depression Inventory at the trial endpoint [36].

Thus, the aim of this study was to compare adjunctive aspirin and rosuvastatin with placebo in youth depression. The primary hypothesis was that after 12 weeks of treatment, both the rosuvastatin and aspirin treatment groups would show greater improvement in depressive symptoms from baseline, compared with the placebo group, on the Montgomery-Åsberg Depression Rating Scale (MADRS) [37]. Secondary hypotheses were that the rosuvastatin and aspirin treatment groups would show greater improvement, compared with the placebo group, on measures of clinical global status, functioning, quality of life, and symptomatology, from baseline to week 12, and that these effects, and the reduction in MADRS, would also be seen at a medium-term week 26 follow-up.

## Methods

### Study design

The study was a 12-week, parallel group, triple-blind, randomised controlled trial (RCT) in participants with moderate to severe MDD. Participants were allocated to receive either rosuvastatin, aspirin, or placebo in statistician-generated sequentially numbered packs in addition to treatment as usual, which usually included psychotherapy or antidepressants. Assessments were completed at baseline and weeks 4, 8, and 12, with a telephone follow-up assessment at week 26 to determine post-discontinuation effects. The study was approved by the Melbourne Health Human Research Ethics Committee (#HREC/12/MH/148). The full protocol was registered on the Australian New Zealand Clinical Trials Registry (ACTRN12613000112763) and is published elsewhere [38].

### Study setting

The study was conducted at six centres in Australia: at the Youth Mood Clinic at Orygen Youth Health in Melbourne, at Jigsaw in Geelong, and across four *headspace* centres in Geelong and north-west Melbourne (Sunshine, Glenroy, Werribee). Treatment as usual at these sites included case management, cognitive behavioural therapy, and pharmacotherapy as per clinician and patient choice. The study ran between June 2013 and June 2017.

### Inclusion and exclusion criteria

The inclusion criteria are as follows: (i) aged between 15 and 25 years; (ii) diagnosis of current MDD, verified using the Structured Clinical Interview for DSM-IV Axis I Disorders, patient version (SCID-I/P) [39]; (iii) MADRS [37] score of 20 or greater, indicating moderate to severe depression; (iv) the ability to give informed consent and to comply with standard procedures; (v) use of effective contraception if female and sexually active with members of the opposite sex; (vi) sufficient fluency in English; and (vii) stable pharmacological treatment for at least 2 weeks prior to enrolment (changes to medication dose or frequency of therapy excepted) if currently being treated.

The exclusion criteria are as follows: (i) lifetime or current SCID-I/P diagnosis of a psychotic disorder; (ii) lifetime SCID-I/P diagnosis of bipolar I or II disorder or alcohol dependence; (iii) acute or unstable systemic medical disorder; (iv) inability to comply with the requirements of informed consent or the study protocol; (v) history of intolerance or allergy to study medications; (vi) current pregnancy or breast feeding; (vii) current regular use of statins, aspirin, non-steroidal anti-inflammatory drugs, paracetamol, corticosteroids, or any other immunomodulatory agents; and (viii) current or recent use of hypolipidemics, vitamin K antagonists and other anticoagulants, protease inhibitors, ketoconazole, spironolactone, or cimetidine.

### Discontinuation and withdrawal

Discontinuation of a participant could be at the discretion of the participant, researcher, or treating physician. Automatic discontinuation occurred if a participant developed a psychotic disorder or bipolar disorder, became pregnant, or was no longer using effective contraception, or if they commenced rosuvastatin or aspirin treatment. Due to the increased risk of myopathy with rosuvastatin and concurrent heavy alcohol use, a score > 20 on the Alcohol Use Disorders Identification Test (AUDIT) [40] necessitated review by the participant’s treating physician, and potential discontinuation. When participants withdrew their consent from the study, all study involvement was ceased but their data was included in the study.

### Interventions

In addition to treatment as usual, participants received either 10 mg/day rosuvastatin, 100 mg/day aspirin, or placebo. At each visit, participants were requested to return all unused investigational products. Adherence to medication was assessed by a pill count, completed by the unblinded study monitor and the clinical trials pharmacist.

The doses of rosuvastatin and aspirin were derived from literature describing the doses at which the agents’ targeted actions are effective and safe [25, 41]. The 10-mg rosuvastatin dose reflects the lowest prescribed therapeutic dose [42]. The 100-mg dose of aspirin is the typical dose used to prevent cardiac events and has been shown to have anti-inflammatory properties [42]. All tablets were over-encapsulated for blinding purposes, in order to be identical in appearance and taste.

### Outcome measures

Changes in the following measures were used to assess efficacy: the interviewer-rated MADRS [37] (primary outcome measure), the Quick Inventory of Depression Symptomatology–Self Report (QIDS-SR) [43], the Generalised Anxiety Disorder seven-item scale (GAD-7) [44], the Clinical Global Impression-Improvement/Severity scale [45] (CGI-I/S), and the self-rated global symptoms, assessed using the Patient Global Impression Improvement (PGI-I) [46]. Quality of life and functioning was assessed at baseline and week 12 using the Quality of Life Enjoyment and Satisfaction Questionnaire–Short Form (Q-LES-Q-SF) [47] and the Social Adjustment Scale–Self Report (SAS-SR), respectively [48]. The Social and Occupational Functioning Scale (SOFAS) [49] was used to measure psychosocial functioning at baseline and weeks 12 and 26.

### Other measures

Potential predictors and moderators of treatment response were assessed using the Dimensional Assessment of Personality Pathology Basic Questionnaire (DAPP-BQ) [50], the SCID-I/P substance use module [39], the AUDIT [40], and the Negative Problem Orientation Questionnaire (NPOQ) [51]. Although participants with syndromal bipolar disorder (BD) were excluded from the study, possible treatment emergent or subthreshold bipolar symptoms [52] were characterised using the Bipolar Spectrum Diagnostic Scale (BSDS) [53] and the Young Mania Rating Scale (YMRS) [54]. At baseline and week 12, routine blood tests were performed for safety purposes. Participants were reviewed by a treating doctor at baseline, 1 week after commencing medication, and at weeks 4, 8, and 12.

### Safety and adverse events

Data monitoring was conducted by a Data and Safety Monitoring Board, the Project Manager, and the Sponsor (Orygen)-appointed Clinical Research Associate. Adverse events were collected using open questions from the time that informed consent was obtained until the end of the 12-week intervention period. After the 12-week intervention period, adverse events were followed up until the adverse event was resolved or until 7 days after trial medication was ceased. All serious adverse events were reported to the relevant regulatory authorities.

Suicidal thinking was assessed with the Suicidal Ideation Questionnaire (SIQ) [55], and suicidality was assessed with the Columbia Suicide Severity Rating Scale (C-SSRS) [56]. If a participant scored 5 on intensity of suicidal ideation in the past month (‘active suicidal ideation with specific plan and intent’), the participant’s continuation in the study was reviewed. If a participant scored 20 or above on the AUDIT at any trial visit, the treating physician was informed and the participant was reviewed.

### Procedure

**Fig. 1:**
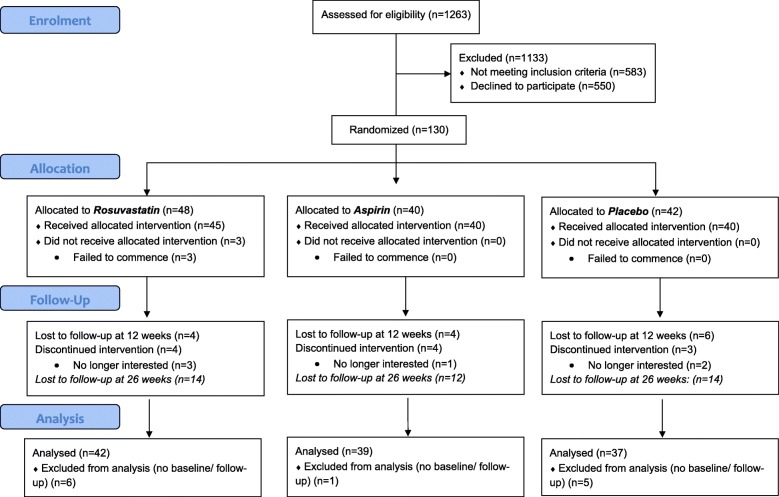
CONSORT diagram of participant flow

Written informed consent was obtained from participants by the investigator or research assistant. If a participant was younger than 18 years old, consent was obtained from both the parent or legal guardian and the participant. Ethics approval was also given for participants aged 16 or 17 to provide their own consent if they were assessed by an independent doctor to be legally competent (a ‘mature minor’). Once eligibility was confirmed, the baseline assessment was conducted (see Fig. 1 for participant flow chart). The participant was then randomly assigned to the rosuvastatin, aspirin, or placebo group on a 1:1:1 basis. Participants, investigators, clinicians, research assistants, and statisticians remained blind to treatment allocation for both data collection and analysis phases (triple blind).

Following baseline assessment and randomisation, participants were assessed at weeks 4, 8, and 12 on measures of psychopathology, functioning, adverse events, and side effects. A follow-up assessment was conducted over the telephone at week 26 exploring the MADRS and SOFAS. For engagement and safety purposes, the participant was also telephoned at week 2. Formal inter-rater reliability assessments were completed annually throughout the study on the primary outcome and key secondary measures such as the SOFAS.

### Randomisation and masking

Participants were randomised according to the International Council for Harmonisation (ICH) Guidelines by an independent researcher, stratified by gender and age (< 18 vs. ≥ 18 years) to the three groups using randomly permuted blocks (6 × 3) to maintain approximately equal group sizes over time in a 1:1:1 ratio, enabled by computer-generated numbers programmed into the electronic case report form (eCRF). Concealed allocation alerts were sent to the local research pharmacists with information regarding participant allocation. The pharmacist and trial coordinator then ensured that study participants received their assigned study treatment. Research coordinators and the clinical team were not aware of allocation. The study biostatistician and others who were involved in preparing the trial results were blinded to intervention allocation. The trial was only unblinded after finalising the analysis. Online unblinding was available for clinical emergencies. If unblinded, participants discontinued treatment in the study but continued to be assessed at the scheduled times, provided they did not withdraw consent.

### Statistical analyses

Primary and secondary analyses were undertaken on intent-to-treat basis, including all participants as randomised, regardless of treatment actually received or their withdrawal from the study, and were reported according to the ICH E9 Statistical Principles for Clinical Trials and Consolidated Standards of Reporting Trials (CONSORT) recommendations [55, 56]. Data collection and entry were conducted according to Good Clinical Practice (GCP) guidelines [57, 58]. No interim analyses were conducted.

Comparisons of those who did and did not complete the follow-up assessments were conducted to identify any bias in missing data; these comparisons were done using one way analysis of variance (ANOVA) and chi-square (*χ*^2^) analyses. All analyses were performed using Stata 15 [59]. The primary efficacy analyses and all secondary continuous outcomes were based on baseline-adjusted mean differences between aspirin and placebo, and rosuvastatin and placebo at week 12. Population average models using a generalised estimating equation (GEE) approach accounting for within-individual repeated measures using a non-specified or exchangeable working correlation matrix were used. The GEE approach was used rather than linear mixed models as the latter involve unverifiable assumptions regarding the data-generating distribution, which can lead to potentially misleading estimates. On the other hand, GEE models with Sandwich estimator are robust to misspecification of the covariance structure. These factors are particularly pertinent given the modest achieved sample size [60, 61]. GEE models contained the fixed effect of intervention allocation and nominal measurement time points as main effects and two-way interaction between intervention allocation and measurement time. The two-way interaction of intervention allocation and measurement time was used to estimate differential change in each intervention compared with placebo.

A priori, planned comparisons of between-group mean change from baseline to the week 12 endpoint were used to test the primary hypothesis (i.e. the two-way interaction between intervention allocation and measurement time at week 12 and its 95% confidence interval). In addition, post hoc between-group comparison of aspirin vs. placebo and rosuvastatin vs. placebo at all other follow-up time points including post-discontinuation assessment at week 26 (estimated from a separate GEE including all measurement time points, including week 26) used the same methods as previously outlined. The comparative effectiveness of aspirin and rosuvastatin was examined on outcome variables as post hoc analyses. Effect size (Cohen’s *d*) was calculated [62]. Remission and response analyses were conducted by dichotomising the MADRS score using cut-off scores of 7 or less for remission, and more than 50% improvement compared with baseline for response, respectively. Logistic regression was used for remission and response analyses. Safety data were compared between treatment arms using Fisher’s exact test.

Additional subgroup analyses for age (< 18 vs. ≥ 18 years), BMI (< 30 vs. ≥ 30), severity using the QIDS (QIDS < 20 vs. QIDS ≥ 20), and number of major depressive episodes (MDD episodes ≤ 2 vs. MDD episodes > 2) were performed. These subgroup analyses were selected based on the literature suggesting that these are potentially predictive demographic characteristics [63–65]. The impact of baseline treatment characteristics including number of concomitant medications during the trial, psychotherapy, or antidepressants was examined from a separate GEE including all measurement time points (excluding week 26) using the same methods as previously outlined. All tests of treatment effects were conducted using an alpha level of 0.05.

### Determination of sample size

A sample size of 270 was estimated to sufficiently power the study (80% power) to detect differences in change from baseline of approximately 0.4 standard deviations in a priori contrasts of treatment arms conducted within the framework of omnibus test of condition-by-time repeated measures ANOVA, accounting for attrition and assuming a correlation of 0.5 between pre- and post-test measurements.

## Results

### Participant characteristics

Participants were recruited and followed up between July 2013 and August 2017. A total of 1263 potential participants were considered for eligibility for the trial (including pre-screening and formal study screening), of which 130 were randomised to receive aspirin (*n* = 40), rosuvastatin (*n* = 48), or placebo (*n* = 42). A total of 109 participants completed the treatment phase and follow-up assessment at week 12: aspirin (*n* = 35), rosuvastatin (*n* = 40), and placebo (*n* = 34), and 81 completed the post-discontinuation assessment at week 26: aspirin (*n* = 25), rosuvastatin (*n* = 30), and placebo (*n* = 26). The CONSORT flow chart (Fig. 1) illustrates participant flow. Participants did not differ significantly on any baseline demographics across the study’s three arms (Table 1).

**Table 1:** Baseline demographic characteristics of participants randomized to rosuvastatin, aspirin, or placebo^a^

Variable	Overall sample (*N* = 130)	Rosuvastatin (*n* = 48)	Aspirin (*n* = 40)	Placebo (*n* = 42)
	Mean (S_D_)	Mean (S_D_)	Mean (S_D_)	Mean (S_D_)
Age (years)	20·2 (2·6)	20·0 (2·7)	20·7 (2·7)	20·0 (2·4)
BMI	25·8 (7·6)	24·3 (6·9)	25·3 (8·1)	27·6 (7·4)
	N (%)	N (%)	N (%)	N (%)
Gender				
Female	77 (59·2)	28 (58·3)	25 (62·5)	24 (57.1)
Age (younger than 18)	29 (22·3)	13 (27·1)	8 (20·0)	8 (19·0)
English first language	119 (91·5)	44 (91.7)	38 (95·0)	37 (88·1)
Occupation^b^				
Student	55 (42·3)	22 (45·8)	16 (40·0)	17 (40·5)
Full-time employment	8 (6·2)	3 (6·3)	3 (7·5)	2 (4·8)
Part time/ Casual	36 (27·7)	14 (29·2)	11 (27·5)	11 (26·2)
unemployed	28 (21·5)	8 (16·7)	11 (27·5)	9 (21·4)
Living status^c^				
Parents	82 (63·1)	25 (52·1)	26 (65·0)	31 (73·8)
Friends	16 (12·3)	8 (16·7)	4 (10·0)	4 (9·5)
Spouse/ De facto	12 (9·2)	5 (10·4)	6 (15·0)	1 (2·4)
Alone	5 (3·8)	3 (6·3)	2 (5·0)	0 (0·0)
Psychosocial therapy during lifetime (yes)	111 (85·4)	40 (83·3)	34 (85·0)	37 (88·1)
Ongoing psychological therapy during study (yes)	90 (69.2)	31 (64.6)	31 (77.5)	28 (66.7)
Antidepressant use (yes)	109 (83·0)	37 (77·1)	35 (87·5)	37 (88·1)
Ongoing antidepressant use during study (yes)	39 (30·0)	13 (27·1)	14 (35·0)	12 (28·6)
CSSRS – past month				
Suicidal ideation	124 (95·4)	46 (95·8)	38 (95·0)	40 (95.2)
Suicidal behaviors	10 (7·7)	3 (6·3)	5 (12·5)	2 (4·8)
Suicidal acts	6 (4·6)	2 (4·2)	3 (7·5)	1 (2·4)
Non-suicidal self-injury	23 (17·7)	10 (20·8)	6 (15·0)	7 (16·7)
Number of major				
Depressive episodes	3 (1, 6)	3 (3, 6)	2 (1, 6)	4 (2, 8)
Duration, weeks	24 (12, 52)	20 (20, 44)	32 (16, 80)	26 (12, 39)
Anxiety disorder	81 (62·3)	31 (64·6)	28 (70·0)	22 (52·4)
Substance use disorder	17 (13·1)	7 (14·6)	4 (10·0)	6 (14·3)
Number of concomitant medications (median, IQR)	3 (2, 5)	3 (2, 4·25)	3 (2, 5)	4.5 (2, 6)

^a^There were no significant differences between groups on any variable

^b^Other occupations include caregiver (*n* = 1), and volunteer work (*n* = 1)

^c^Other living status include other relatives (*n* = 2) and other arrangements (*n* = 10)

On average, the participants had severe depressive symptoms, with mean baseline MADRS scores of 32.6 (± 6.1) in rosuvastatin, 32.6 (± 5.5) in aspirin, and 32.3 (± 6.5) in the placebo group. They had high levels of co-morbid anxiety disorders (present in 62.8%) and past-month suicidal ideation (96.1%), indicative of their help-seeking status and relatively severe presentations, and they had experienced a median of three major depressive episodes (Table 1).

### Primary outcomes

**Fig. 2:**
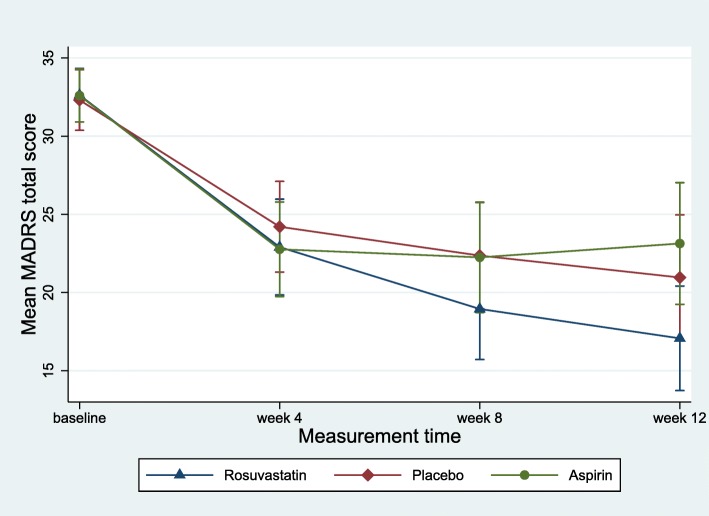
Margins plot showing change in MADRS score (with whiskers representing 95% CI) from baseline to 4 week, 8 week, and 12 week follow-ups in rosuvastatin, aspirin, and placebo

Results of the GEE analysis for the primary outcomes of depression symptoms (MADRS) are summarised in Table 2. Considering all post-baseline time points during the trial treatment phase, there were no significant differential changes in levels of depression symptoms between rosuvastatin and placebo (${\chi}_3^2$ = 3.7, *p* = 0.296), or between aspirin and placebo (${\chi}_3^2$ = 2.5, *p* = 0.468—Fig. 2). A priori comparison of change from baseline to week 12 showed − 4.2 (95% CI [− 9.1, 0.6]) additional improvement (i.e. change from baseline to week 12) in rosuvastatin, compared with placebo; however, the difference was not statistically significant (*p* = 0.089). There was no significant separation between aspirin and placebo (*p* = 0.433) on the MADRS. The rosuvastatin vs. aspirin group comparison was statistically significant across all post-baseline time points (${\chi}_3^2$ = 8.6, *p* = 0.035); post hoc pairwise comparisons also revealed significant differences at week 12 (*p* = .017) in favour of rosuvastatin, compared with aspirin.

**Table 2:** Primary outcome measures comparing rosuvastatin and aspirin to placebo, with 12 weeks follow-up as the primary comparison

	Rosuvastatin *(n = 48)*	Aspirin *(n = 40)*	Placebo* (n = 42)*	Rosuvastatin vs placebo			Aspirin vs placebo			Rosuvastatin vs aspirin
	Mean (SD)			*p* value	Differential change^b^ (95% CI)	Effect size^c^	*p* value	Differential change^b^ (95% CI)	Effect size^c^	Differential change^b^ (95% CI)
MADRS (depression)										
Overall^a^	–	–	–	0.296	–	–	0.467	–	–	
Baseline	32.6 (6.1)	32.6 (5.5)	32.3 (6.5)							
4 weeks	23.1 (10.2)	22.7 (9.7)	24.0 (9.2)		− 1.6 (− 5.7, 2.5)	− 0.21	0.357	− 1.7 (− 5.4, 2.0)	− 0.24	0.0 (− 4.3, 4.3)
8 weeks	19.1 (10.7)	22.0 (11.1)	22.1 (10.6)		− 3.7 (− 8.1, 0.6)	− 0.43	0.854	− 0.4 (− 4.7, 3.9)	− 0.07	3.5 (− 8.3, 1.2)
12 weeks	17.2 (11.0)	22.9 (12.0)	20.4 (12.4)	0.089	− 4.2 (− 9.1, 0.6)	− 0.44	0.433	1.9 (− 2.8, 6.6)	0.16	− 6.4 (− 11.7, 1.2)
26 weeks^d^	16.0 (12.3)	15.7 (10.4)	13.2 (10.4)	0.487	1.9 (− 3.4, 7.2)	0.12	0.353	2.5 (− 2.7, 7.6)	0.10	0.3 (− 6.0, 6.7)

*MADRS* Montgomery-Asberg Depression Rating Scale

^a^Intervention by follow-up interaction test

^b^Two-way interaction of intervention allocation and measurement time (baseline adjusted between group mean difference estimated from GEE model)

^c^Cohen’s *d* effect size

^d^From a GEE model that includes baseline and week 4 to week 26 measures

### Secondary outcomes

Results of the GEE analysis on the secondary outcomes are summarised in Additional file 1: Table S3. No significant between-group differences were observed in QIDS-SR, SAS-SR, CGI-I, PGI, Social and Occupational Functioning Scale (SOFAS), or Generalised Anxiety Disorder Scale (GAD-7) scores over the 12-week trial in either the rosuvastatin or the aspirin groups compared with placebo. At the week 26 post-discontinuation follow-up, there were no significant differences between placebo and either rosuvastatin or aspirin. The rosuvastatin group had a greater reduction in the NPOQ compared with the aspirin group at week 12 (*p* = 0.025), but there was no significant difference between aspirin or rosuvastatin compared with placebo groups.

The CGI-S was significantly reduced in the rosuvastatin group, compared with the aspirin group at week 12, but there was no significant improvement in the rosuvastatin or aspirin group when compared with placebo. Quality of life, as measured by the Q-LES-Q-SF, increased significantly less (*p* = 0.04) in the aspirin group, compared with placebo at week 12. Baseline treatment characteristics such as number of concomitant medications during the trial, psychotherapy, or history/ongoing use of antidepressants did not significantly affect these results (Additional file 1: Table S5 and S6).

### Response and remission

The 12-week MADRS response rates were 45.8%, 25.0%, and 33.3% in the rosuvastatin, aspirin, and placebo group, respectively (${\chi}_2^2$ = 4.3, *p* = 0.119), and MADRS remission rates at week 12 were 15.0%, 15.2%, and 15.2%, in the rosuvastatin, aspirin, and placebo group, respectively (${\chi}_2^2$ = 0.0, *p* > .999).

### Safety and adverse events

Suicidal thinking was assessed using SIQ ≥ 41 as the cut-off. There were no significant differences between the aspirin and rosuvastatin groups, compared with placebo (Additional file 1: Table S3). There was a significant reduction in the AUDIT (Additional file 1: Table S3) in both the aspirin and rosuvastatin groups, compared with placebo, at weeks 4 and 8 (*p* < 0.05 and Cohen’s *d* > 0.5), though not at week 12.

The frequency and percentage of adverse events among participants, along with their severity (severe, moderate, or mild), are presented in Additional file 1: Table S1. The rates were similar across the trial arms, and there were no significant differences in adverse event rates (Additional file 1: Table S1). A significantly higher rate of any concomitant medication was used in the placebo group (71.4% in the placebo group vs. 42.1% in the rosuvastatin group, and 44.0% in the aspirin group, *p* < 0.05). Four participants in the aspirin group withdrew as a result of adverse events (bleeding, muscle weakness, psychotic episode, and sinusitis), compared with only one participant in the rosuvastatin group (hospitalisation due to major depression episode), and no participant in the placebo group (*p* < 0.05). The most frequent adverse events in each of the trial arms are shown in Additional file 1: Table S2.

### Subgroup analyses

Three subgroup analyses by age (≤ 18 years, and > 18 years), BMI (BMI < 30, and BMI ≥ 30), severity indexed by baseline QIDS (< 20, and ≥ 20), and number of MDD episodes (≤ 2, and > 2) were performed on the MADRS. In the age ≤ 18 years subgroup (Additional file 1: Table S3), there was a significant reduction in depressive symptoms in the rosuvastatin group compared with the placebo group at week 8 (*p* = 0.025; Cohen’s *d*, 0.9; MADRS improvement − 8.7; 95% CI (− 16.3, − 1.1)) and the week 12 endpoint (*p* = 0.029; Cohen’s *d*, 1.1; MADRS improvement − 9.8; 95% CI (− 17.7, − 1.0)). While there was no significant difference in the aspirin group compared with the placebo group at week 12, there was greater improvement in MADRS scores at weeks 4 and 8 in the aspirin group (Cohen’s *d*, 1.2 and 1.2, respectively). There were no significant between-group differences on the MADRS for participants > 18.

There were no significant differences in MADRS outcome in the BMI subgroup analyses (Additional file 1: Table S4). In the baseline severity (QIDS ≥ 20) subgroup analysis, there was a significant improvement (*p* = 0.045) at week 12 on the MADRS in the rosuvastatin group compared with placebo (Additional file 1: Table S4; Cohen’s *d*, 0.8). In the number of MDD episodes (number of episodes > 2) subgroup analysis, there was a significant improvement (*p* = 0.045) at week 12 on the MADRS in the rosuvastatin group compared with placebo (Cohen’s *d*, 0.7). There were no significant between-group differences on the MADRS measure for people who had ≤ 2 episodes.

## Discussion

The primary hypothesis that each of the rosuvastatin and aspirin treatment groups would show greater improvement, compared with placebo, was not supported. Specifically, no significant between-group differences were found on the a priori primary outcome (MADRS score) at the primary endpoint (week 12), although the difference between rosuvastatin and placebo at week 12 narrowly missed significance (*p* = 0.089). There were no significant differences on other outcome measures in the rosuvastatin group compared with placebo. Some positive pre-specified secondary findings were in favour of rosuvastatin, principally the difference between rosuvastatin and aspirin on the MADRS. Concordant with this, rosuvastatin was superior to aspirin on depression assessed on the MADRS, global clinical severity, and dysfunctional attitudes toward social problem-solving using the Negative Problem Orientation Questionnaire scale (NPOQ). Nevertheless, there were no differences in remission rates between groups. While remission rates were similar across treatment groups, response rate ranged from 25 to 45.8%. In 30 cases (5 cases in aspirin, 16 cases in rosuvastatin, and 9 cases in placebo), while there were more than 50% reduction from baseline at week 12 (MADRS response), the absolute value of MADRS score at week 12 was more than 7 (i.e. no remission). Baseline MADRS score for these 30 cases was 32.3 (5.6). Together with higher use of concomitant medication in the placebo group, these findings provide a tentative suggestion for potential utility of statins, but indicate a lack of efficacy of low dose aspirin.

Exploratory subgroup analyses showed an effect of age, with an efficacy signal in younger (age ≤ 18 years) participants in the rosuvastatin group, but as with all non-primary findings, this should be interpreted with considerable caution. If this holds up to replication, it might suggest differential treatment approaches, contingent on age. Similarly, there was an effect of greater baseline depression severity (QIDS ≥ 20) predicting response in the rosuvastatin group, concordant with antidepressant studies showing greater efficacy in more unwell participants.

Strengths of the study include its pragmatic and real-world setting, focus on youth-specific health facilities, the homogeneity of the cohort, the tolerability of the study agents, and the high external validity of the trial design, reflected in the relative lack of exclusion criteria to reflect add-on to routine care. It is generally more difficult to demonstrate add-on efficacy than for monotherapy: notably, 84.5% of the sample had a prior history of antidepressant use, while 39% were taking antidepressants while in the trial. The sample size was relatively small and was lower than planned recruitment based on the power analyses due to governance delay and operational issues, which could result in low statistical power to reliably detect between-group differences. A total of 1133 people who were approached were excluded, principally for not meeting inclusion criteria (*n* = 583) and declining to participate in research (*n* = 550). The pre-specified secondary outcomes were not corrected for multiplicity; however, to mitigate the risk of family-wise multiple comparisons, *p* values were extracted only for the a priori comparisons. In addition, in subgroup analyses, *p* values were extracted only for a priori comparisons at week 12 and week 26 sustainability comparisons. As such, findings need to be interpreted with caution, as some exploratory findings might be spurious.

The relatively low dose of aspirin used might also have been a factor. While most of the epidemiological data explored the use of similar low dose strategies, some successful clinical trials, such as one in schizophrenia, have used a far higher dose [66]—1000 mg daily. Similarly, the choice of statins was guided by their different pharmacodynamic profiles. Our hypothesis was that statins, like aspirin [67], would be efficacious by suppressing peripheral inflammation, and rosuvastatin had the strongest evidence base for suppressing markers of inflammation, hence its use in the study, mindful that both agents have other mechanistic targets [68]. However, one could argue that based on epidemiological and preclinical data [69], lipophilicity might be an important factor, as it determines brain bioavailability, and indeed rosuvastatin is hydrophilic—this is an equipoise issue. Lipophilic statin with blood-brain barrier penetration might be more effective at engaging a central nervous system target. Some, but not all patients, were taking adjunctive antidepressants. The use of severity cut-offs for inclusion risks regression to the mean and inflation of placebo effects. Blood sampling for biomarker associations of treatment effects has been done, but these results will be presented separately.

## Conclusions

In summary, low-dose aspirin does not appear to be more effective than placebo in youth depression. Rosuvastatin was also found not to be more effective than placebo on the primary outcome, but there were signals of efficacy on several secondary measures—which need to be interpreted with caution. Anti-inflammatory agents for depression could be used at low-to-moderate doses when prescribed as augmentation strategies when monoamine-modulating antidepressants do not lead to satisfactory responses. This study provides limited support for the role of rosuvastatin in youth depression, an age cohort where antidepressants are of uncertain value [9], and provides possible proof of principle support for the role of inflammation in this cohort.

## Supplementary information

**Additional file 1: Table S1.** Adverse event data: comparing Rosuvastatin and Aspirin to placebo. **Table S2.** Most frequent adverse events. **Table S3.** Secondary outcome measures comparing Rosuvastatin and Aspirin to placebo; with 12 weeks follow-up as the primary comparison. **Table S4.** Subgroup analyses: comparing Rosuvastatin and Aspirin to placebo; with 12 weeks follow-up as the primary comparison. **Table S5.** History and ongoing psychological therapy: comparing Rosuvastatin and Aspirin to placebo. **Table S6.** History and ongoing antidepressants therapy: comparing Rosuvastatin and Aspirin to placebo.

## Data Availability

The datasets used and/or analysed during the current study are available from the corresponding author on reasonable request.

## Abbreviations

AUDIT: Alcohol Use Disorders Identification Test
BSDS: Bipolar Spectrum Diagnostic Scale
CGI-I/S: Clinical Global Impression-Improvement/Severity scale
CONSORT: Consolidated Standards of Reporting Trials
C-SSRS: Columbia Suicide Severity Rating Scale
DAPP-BQ: Dimensional Assessment of Personality Pathology Basic Questionnaire
eCRF: Electronic case report form
GAD-7: Generalised Anxiety Disorder seven-item scale
GCP: Good Clinical Practice
GEE: Generalised estimating equation
ICH: International Council for Harmonisation
IDO: Indoleamine-pyrrole 2, 3-dioxygenase
LFT: Liver function tests
MADRS: Montgomery-Åsberg Depression Rating Scale
MDD: Major depressive disorder
NPOQ: Negative Problem Orientation Questionnaire Scale
PGI-I: Patient Global Impression Improvement
QIDS-SR: Quick Inventory of Depression Symptomatology–Self Report
Q-LES-Q-SF: Quality of Life Enjoyment and Satisfaction Questionnaire–Short Form
RCT: Randomised controlled trial
SAS-SR: Social Adjustment Scale–Self Report
SCID-I/P: Structured Clinical Interview for DSM-IV Axis I Disorders
SIQ: Suicidal Ideation Questionnaire
SOFAS: Social and Occupational Functioning Scale
U&E: Urea and electrolytes
YMRS: Young Mania Rating Scale
YoDA-A: Youth Depression Alleviation with Anti-inflammatory Agents
YoDA-C: Youth Depression Alleviation-Combined Treatment
