# Supplementary material for: Youth Depression Alleviation with Anti-inflammatory Agents (YoDA-A): a randomised clinical trial of rosuvastatin and aspirin
Source: BMC Med. 2020 Jan 17;18:16. doi: 10.1186/s12916-019-1475-6 (PMC6966789; doi:10.1186/s12916-019-1475-6)
Supplement: Supplementary file 1 — Additional file 1: Table S1. Adverse event data: comparing Rosuvastatin and Aspirin to placebo. Table S2. Most frequent adverse events. Table S3. Secondary outcome measures comparing Rosuvastatin and Aspirin to placebo; with 12 weeks follow-up as the primary comparison. Table S4. Subgroup analyses: comparing Rosuvastatin and Aspirin to placebo; with 12 weeks follow-up as the primary comparison. Table S5. History and ongoing psychological therapy: comparing Rosuvastatin and Aspirin to placebo. Table S6. History and ongoing antidepressants therapy: comparing Rosuvastatin and Aspirin to placebo. [file 12916_2019_1475_MOESM1_ESM.docx]

**Table S1. Adverse event data: comparing Rosuvastatin and Aspirin to placebo**

|  | n (% of participants) | Rosuvastatin | Aspirin | Placebo |
| --- | --- | --- | --- | --- |
| Any adverse event |  | 38 (79·2) | 25 (62·5) | 28 (66·7) |
| Severe adverse event |  | 2 (4·2) | 4 (10·0) | 2 (4·8) |
| Moderate adverse event |  | 15 (31·3) | 13 (32·5) | 10 (23·8) |
| Mild adverse event |  | 37 (77·1) | 20 (50) | 26 (61·9) |
| Causality of adverse event |  |  |  |  |
|  | Probable | 2 (5·3) | 4 (16·0) | 2 (7·1) |
|  | Possible | 20 (52·6) | 7 (28·0) | 12 (42·9) |
|  | Improbable | 16 (42·1) | 14 (56·0) | 14 (56·0) |
| Action taken | Concomitant Medication^*^ | 16 (42·1) | 11 (44·0) | 20 (71·4) |
|  | Non-drug therapy | 4 (10·5) | 1 (4·0) | 2 (7·1) |
|  | Dose interrupted | 1 (2·6) | 1 (4·0) | 1 (3·6) |
|  | Dose permanently stopped^*^ | 1 (2·6) | 4 (16·0) | 0 (0·0) |

*Significant at 0.05 (Aspirin vs placebo)

**Table S2: Most frequent adverse events**

|  | Rosuvastatin | Aspirin | Placebo |
| --- | --- | --- | --- |
| Headache | 11 (26·2) | 4 (10·3) | 8 (21·6) |
| Cold | 7 (16·7) | 2 (5·1) | 7 (18·9) |
| Pain | 5 (11·9) | 2 (5·1) | 5 (13·5) |
| Nausea | 5 (11·9) | 3 (7·7) | 5 (13·5) |
| Fatigue | 2 (4·8) | 2 (5·1) | 2 (5·4) |
| Bruising^*^ | 1 (2·4) | 5 (12·8) | 0 (0) |
| Overdose | 4 (9·5) | 1 (2·6) | 0 (0) |
| Anxiety | 2 (4·8) | 0 (0) | 1 (2·7) |
| Cough | 2 (4·8) | 1 (2·6) | 2 (5·4) |
| Fainted | 1 (2·4) | 1 (2·6) | 1 (2·7) |
| Fever | 3 (7·1) | 1 (2·6) | 0 (0) |
| Flu | 1 (2·4) | 1 (2·6) | 2 (5·4) |
| Self-harm | 3 (7·1) | 1 (2·6) | 0 (0) |
| Vomiting | 2 (4·8) | 0 (0) | 1 (2·7) |
| Bleeding | 0 (0) | 1 (2·6) | 1 (2·7) |
| Suicide ideation | 1 (2·4) | 0 (0) | 1 (2·7) |
| Suicide attempt | 0 (2·4) | 1 (2·6) | 0 (0) |
| Hospitalisation | 0 (0) | 3 (7·7) | 1 (2·7) |

**Table S3. Secondary outcome measures comparing Rosuvastatin and Aspirin to placebo; with 12 weeks follow-up as the primary comparison.**

|  |  |  | Rosuvastatin | Aspirin | Placebo | Rosuvastatin and Placebo | | | | Aspirin and Placebo | | | | Rosuvastatin and Aspirin |
| --- | --- | --- | --- | --- | --- | --- | --- | --- | --- | --- | --- | --- | --- | --- |
|  |  |  | Mean (S_D_) | | | *p*-value | Differential change ^b^ | 95% CI | Effect size^c^ | p-value | Differential change ^b^ | 95% CI | Effect size^c^ | Differential change^b^  (95% CI) |
| YMRS (Young Mania Rating Scale) |  | Baseline | 2·3 (2·2) | 2·7 (2·0) | 3·0 (2·0) |  | | | | | | | | |
|  |  | 12 weeks | 1·6 (1·8) | 2·2 (2·6) | 1·9 (1·5) | 0·667 | 0·2 | (-0·8, 1·3) | 0·0 | 0·311 | 0·6 | (-0·6, 1·9) | 0·3 | -0·8 (-2·1, 0·5) |
| SOFAS (Social and Occupational Functioning Scale) |  | Baseline | 57·1 (10·6) | 58·0 (12·6) | 57·5 (11·4) |  | | | | | | | | |
|  |  | 12 weeks | 67·7 (13·6) | 60·9 (16·7) | 64·1 (17·0) | 0·157 | 4·3 | (-1·7, 10·1) | 0·4 | 0·367 | -3·9 | (-9·4, 3·5) | -0·2 | 6·9 (0·9, 12·9) |
|  |  | 26 weeks^d^ | 70·5 (11·5) | 64·4 (17·9) | 68·5 (17·2) | 0·455 | 2·8 | (-4·5, 10·0) | 0·1 | 0·291 | -4·7 | (-13·5, 4·0) | -0·3 | 7·4 (-0·8, 15·7) |
| Q-LES-Q-SF |  | Baseline | 35·0 (14·6) | 40·1 (15·1) | 34·6 (10·7) |  | | | | | | | | |
|  |  | 12 weeks | 45·5 (16·9) | 45·0 (21·2) | 50·0 (19·5) | 0·271 | -4·6 | (-12·7, 3·6) | -0·2 | **0**·**040** | -9·8 | (-19·3, -0·4) | -0·5 | 4·7 (-3·8, 13·2) |
| QIDS-SR (Quick Inventory of Depression Symptomatology-Self Report) |  | Overall^a^ | - | - | - | 0·581 | - | - | - | 0·868 | - | - | - | - |
|  |  | Baseline | 17·2 (4·7) | 16·3 (3·6) | 17·7 (3·6) |  | | | | | | | | |
|  |  | 4 weeks | 12·9 (5·0) | 12·5 (4·4) | 13·8 (4·8) |  | -0·3 | (-2·4, 1·9) | -0·0 | 0·978 | 0·0 | (-2·0, 2·0) | 0·0 | -0·3 (-2·5, 2·0) |
|  |  | 8 weeks | 12·3 (5·4) | 12·1 (4·5) | 12·2 (4·9) |  | 0·3 | (-2·1, 2·7) | -0·0 | 0·510 | 0·8 | (-1·6, 3·2) | 0·1 | -0·6 (-3·2, 2·1) |
|  |  | 12 weeks | 11·0 (5·8) | 11·7 (5·3) | 12·3 (5·6) | 0·407 | -1·1 | (-3·7, 1·5) | -·2 | 0·674 | 0·5 | (-2·0, 3·1) | 0·1 | -1·7 (-4·5, 1·1) |
| SIQ≥41  (Suicidal Ideation Questionnaire) ^e^ |  | Overall^a^ | - | - | - | 0·431 | - | - | - | 0·425 | - | - | - | - |
|  |  | Baseline | 37 (82·2) | 33 (84·6) | 31 (79·5) |  | | | | | | | | |
|  |  | 4 weeks | 30 (73·2) | 23 (65·7) | 25 (71·4) |  | 0·7 | (0·2, 2·3) | -0·2 | 0·957 | 1·0 | (0·4, 4·2) | 0·0 | 0·6 (0·2, 2·3) |
|  |  | 8 weeks | 24 (66·7) | 20 (62·5) | 17 (53·1) |  | 0·9 | (0·3, 2·6) | -0·1 | 0·167 | 2·2 | (0·7, 6·6) | 0·4 | 0·4 (0·2, 1·1) |
|  |  | 12 weeks | 19 (50·5) | 20 (62·5) | 15 (48·4) | 0·167 | 0·6 | (0·2, 1·3) | -0·3 | 0·968 | 1·0 | (0·4, 2·6) | 0·0 | 0·5 (0·2, 1·4) |
| NPOQ |  | Baseline | 39·2 (11·5) | 37·4 (11·0) | 39·4 (8·8) |  | | | | | | | | |
|  |  | 12 weeks | 33·4 (12·6) | 35·5 (9·7) | 34·3 (10·5) | 0·948 | -0·2 | (-5·0, 4·7) | -0·0 | 0·093 | 4·3 | (-0·7, 9·3) | 0·5 | -4·8 (-8·9, 0·6) |
| GAD-7 (Generalized Anxiety Disorder 7-item scale) |  | Overall^a^ | - | - | - | 0·758 | - | - | - | 0·829 | - | - | - | - |
|  |  | Baseline | 12·4 (6·3) | 12·3 (4·9) | 13·3 (4·4) |  | | | | | | | | |
|  |  | 4 weeks | 9·0 (5·5) | 10·1 (6·0) | 10·3 (4·9) |  | -0·6 | (-3·1, 1·9) | -0·1 | 0·610 | 0·6 | (-1·7, 3·0) | 0·1 | -1·2 (-3·9, 1·5) |
|  |  | 8 weeks | 9·7 (5·9) | 9·5 (6·2) | 9·9 (4·4) |  | 0·3 | (-2·2, 2·9) | 0·0 | 0·445 | 1·0 | (-1·6, 3·7) | 0·2 | -1·1 (-4·1, 1·8) |
|  |  | 12 weeks | 8·1 (6·4) | 9·6 (6·2) | 9·6 (6·4) | 0·684 | -0·6 | (-1·7, 3·0) | -0·1 | 0·358 | 1·4 | (-1·5, 4·3) | 0·3 | -2·2 (-4·8, 0·5) |
| AUDIT (Alcohol Use Disorders Identiﬁcation Test) |  | Overall^a^ | - | - | - | **0·044** | - | - | - | 0·081 | - | - | - | - |
|  |  | Baseline | 7·9 (5·7) | 7·2 (6·0) | 8·0 (5·8) |  | | | | | | | | |
|  |  | 4 weeks | 5·8 (4·2) | 5·6 (4·0) | 8·2 (5·9) |  | -2·2 | (-4·2, -0·2) | -0·6 | **0·017** | -2·6 | (-4·7, -0·5) | -0·6 | 0·3 (-2·0, 2·5) |
|  |  | 8 weeks | 4·7 (3·9) | 4·2 (2·6) | 7·3 (5·2) |  | -3·5 | (-5·9, -1·0) | -0·8 | **0·027** | -2·5 | (-4·8, -0·3) | -0·8 | -1·3 (-4·9, 2·2) |
|  |  | 12 weeks | 6·5 (4·3) | 4·8 (4·1) | 6·0 (4·1) | 0·182 | -1·7 | (-4·2, 0·8) | -0·5 | 0·391 | -1·1 | (-3·6, 1·4) | -0·4 | -0·7 (-4·1, 2·8) |
| CGI-S (Clinical Global Impression-Severity scale) |  | Overall^a^ | - | - | - | 0·261 | - | - | - | 0·631 | - | - | - | - |
|  |  | Baseline | 4·7 (0·7) | 4·3 (0·8) | 4·6 (0·7) |  | | | | | | | | |
|  |  | 4 weeks | 3·7 (1·0) | 3·9 (1·0) | 3·9 (0·8) |  | -0·3 | (-0·7, 0·1) | -0·3 | 0·216 | 0·2 | (-0·1, 0·1) | 0·2 | -0·5 (-1·0, -0·0) |
|  |  | 8 weeks | 3·3 (1·3) | 3·5 (1·1) | 3·4 (1·0) |  | -0·2 | (-0·7, 0·3) | -0·2 | 0·319 | 0·3 | (-0·3, 0·8) | 0·2 | -0·5 (-1·1, 0·2) |
|  |  | 12 weeks | 2·9 (1·3) | 3·4 (1·1) | 3·4 (1·3) | 0·082 | -0·5 | (-1·1, 0·1) | -0·4 | 0·357 | 0·3 | (-0·3, 0·9) | 0·2 | -0·8 (-1·4, -0·1) |
| CGI-I^f^ (Clinical Global Impression-Improvement scale) |  | Overall^g^ | - | - | - | 0·530 | - | - | - | 0·271 | - | - | - | - |
|  |  | 4 weeks | 2·9 (1·2) | 3·2 (1·2) | 3·2 (2·7) |  | -0·2 | (-0·7, 0·3) | -0·2 | 0·819 | 0·1 | (-0·5, 0·6) | 0·1 | -0·3 (-0·8, 0·3) |
|  |  | 8 weeks | 2·8 (1·3) | 3·0 (1·3) | 2·7 (1·1) |  | 0·1 | (-0·5, 0·6) | 0·0 | 0·335 | 0·3 | (-0·3, 0·9) | 0·2 | -0·2 (-0·9, 0·4) |
|  |  | 12 weeks | 2·4 (1·1) | 2·9 (1·2) | 2·7 (1·1) | 0·477 | -0·2 | (-0·8, 0·4) | -0·2 | 0·492 | 0·2 | (-0·4, 0·8) | 0·2 | -0·4 (-1·0, 0·1) |
| PGI^f^ |  | 12 weeks | 3·0 (1·1) | 3·1 (1·1) | 3·3 (1·5) | 0·210 | -0·4 | (-1·0, 0·2) | -0·3 | 0·525 | -0·2 | (-0·8, 0·4) | -0·2 | -0·2 (-0·7, 0·4) |
| BSDS^f^ |  | 4 weeks | 13·6 (3·5) | 13·6 (2·9) | 13·2 (3·3) | 0·692 | 0·4 | (-1·2, 1·8) | 0·1 | 0·793 | 0·4 | (-1·2, 2·0) | 0·1 | -0·1 (-1·6, 1·4) |
| SAS-SR |  | Baseline | 2·9 (0·6) | 2·8 (0·5) | 2·9 (0·5) |  | | | | | | | | |
|  |  | 12 weeks | 2·7 (0·7) | 2·7 (0·5) | 2·5 (0·6) | 0·824 | 0·0 | (-0·2, 0·4) | 0·0 | 0·177 | 0·2 | (-0·1, 0·4) | 0·7 | -0·7 (-1·6, 0·2) |

BSDS: Bipolar Spectrum Diagnostic Scale, SAS-SR: Social Adjustment Scale-Self Report, NPOQ: Negative Problem Orientation Questionnaire, PGI: Patient Global Impression Improvement

^a^ Intervention by follow-up interaction test
^b^ Two-way interaction of intervention allocation and measurement time (between group differential change estimated from GEE)
^c^ Cohen’s *d* effect size
^d^ From a GEE that includes baseline, and week 4 to week 26 measures
^e^ number (percent) is presented
^f^ One-way ANOVA test
^g^ Overall main effect test with placebo group as reference category

**Table S4. Subgroup analyses: comparing Rosuvastatin and Aspirin to placebo; with 12 weeks follow-up as the primary comparison**

| **AGE** |  | **Rosuvastatin** | **Aspirin** | **Placebo** | **Rosuvastatin and Placebo** | | | | | | | **Aspirin and Placebo** | | | | | | | **Rosuvastatin and Aspirin** |
| --- | --- | --- | --- | --- | --- | --- | --- | --- | --- | --- | --- | --- | --- | --- | --- | --- | --- | --- | --- |
|  |  | Mean (S_D_) | | | *p*-value | | Differential change ^b^ | | 95% CI | Effect size^c^ | | *p*-value | | Differential change ^b^ | 95% CI | | Effect size^c^ | | Differential change^b^  (95% CI) |
| MADRS  (≤18 years) (n=26) | Overall^a^ | - | - | - | 0·048 | | - | | - | - | | 0·011 | | - | - | | - | |  |
|  | Baseline | 32·7 (5·4) | 35·4 (6·5) | 31·9 (5·5) |  | | | | | | | | | | | | | |  |
|  | 4 weeks | 21·6 (11·6) | 22·8 (12·0) | 26·0 (8·5) |  | | -5·6 | | (-12·3, 1·1) | -0·6 | |  | | -6·8 | (-12·0, -1·5) | | -1·2 | | 1·2 (-6·3, 8·7) |
|  | 8 weeks | 19·5 (11·9) | 20·7 (12·8) | 27·3 (2·9) |  | | -8·7 | | (-16·3, -1·1) | -0·9 | |  | | -9·8 | (-17·8, -2·0) | | -1·2 | | 1·1 (-7·4, 9·7) |
|  | 12 weeks | 13·7 (10·9) | 22·0 (14·5) | 21·7 (12·5) | **0·029** | | -9·8 | | (-17·7, -1·0) | -1·1 | | 0·632 | | -2·3 | (-11·7, 7·1) | | -0·2 | | -6·9 (-15·1, 1·3) |
|  | 26 weeks^d^ | 11·7 (14·2) | 13·4 (14·3) | 14·4 (12·2) | 0·762 | | -1·9 | | (-14·3, 10·5) | -0·3 | | 0·997 | | 0·0 | (-14·3, 14·2) | | -0·1 | | -1·9 (-14·9, 11·0) |
| MADRS  (>18 years) (n=92) | Overall^a^ | - | - | - | 0·671 | | - | | - | - | | 0·633 | | - | - | | - | |  |
|  | Baseline | 32·9 (6·1) | 31·9 (5·1) | 32·4 (6·8) |  | | | | | | | | | | | | | |  |
|  | 4 weeks | 23·6 (9·9) | 22·6 (9·3) | 23·5 (9·5) |  | | -0·6 | | (-5·5, 4·2) | -0·1 | |  | | 0·5 | (-4·8, 3·9) | | 0·1 | | -0·0 (-5·0, 4·6) |
|  | 8 weeks | 19·0 (10·5) | 22·4 (10·8) | 21·0 (11·4) |  | | -2·9 | | (-7·9, 2·2) | -0·3 | |  | | -1·8 | (-3·1, 6·6) | | -0·2 | | -4·8 (-10·0, 0·5) |
|  | 12 weeks | 18·6 (10·9) | 23·1 (11·5) | 20·1 (12·5) | 0·372 | | -2·7 | | (-8·5, 3·2) | -0·3 | | 0·277 | | -3·0 | (-2·4, 8·3) | | -0·2 | | -5·6 (-11·6, 0·3) |
|  | 26 weeks^d^ | 17·3 (11·6) | 16·3 (9·6) | 12·9 (10·2) | 0·337 | | 2·9 | | (-3·0, 8·9) | 0·2 | | 0·265 | | 3·1 | (2·3, -8·5) | | 0·2 | | -0·0 (-6·5, 6·1) |
| **BMI** |  | **Rosuvastatin** | **Aspirin** | **Placebo** | **Rosuvastatin and Placebo** | | | | | | | **Aspirin and Placebo** | | | | | | | **Rosuvastatin and Aspirin** |
|  |  | Mean (S_D_) | | | *p-*value | Differential change ^b^ | | 95% CI | | | Effect size^c^ | *p*-value | Differential change ^b^ | | | 95% CI | | Effect size^c^ | Differential change^b^  (95% CI) |
| MADRS  (BMI<30)  (n=81) | Overall^a^ | - | - | - | 0·233 | - | | - | | | - | 0·673 | - | | | - | | - |  |
|  | Baseline | 33·7 (5·7) | 33·6 (5·4) | 33·3 (6·2) |  |  | | | | | | | | | | | |  |  |
|  | 4 weeks | 23·0 (10·9) | 23·4 (10·4) | 24·8 (9·5) |  | -2·3 | | (-7·8, 3·1) | | | -0·2 |  | 1·8 | | | (-3·0, 6·6) | | 0·20 | -0·5 (-5·7, 4·7) |
|  | 8 weeks | 17·1 (12·2) | 20·6 (12·3) | 20·8 (12·8) |  | -4·7 | | (-10·2, 0·8) | | | -0·5 |  | 0·9 | | | (-4·3, 6·1) | | 0·07 | -3·9 (-9·4, 1·6) |
|  | 12 weeks | 16·3 (12·0) | 22·4 (14·0) | 20·3 (12·3) | 0·057 | -6·3 | | (-12·7, 0·2) | | | -0·6 | 0·618 | -1·5 | | | (-7·6, 4·5) | | -0·14 | -7·8 (-14·3, -1·3) |
|  | 26 weeks^d^ | 14·4 (12·2) | 16·4 (11·7) | 11·9 (12·9) | 0·656 | 1·6 | | (-5·4, 8·6) | | | 0·0 | 0·231 | 4·5 | | | (-2·8, 11·8) | | 0·19 | -2·9 (-10·2, 4·4) |
| MADRS  (BMI≥30) (n=23) | Overall^a^ | - | - | - | 0·679 | - | | - | | | - | 0·849 | - | | | - | | - |  |
|  | Baseline | 32·3 (6·5) | 29·6 (4·3) | 31·5 (5·2) |  |  | | | | | | | | | | | |  |  |
|  | 4 weeks | 21·1 (9·7) | 21·6 (8·8) | 25·2 (7·6) |  | -5·0 | | (-13·2, 3·1) | | | -0·64 |  | 1·9 | | | (-5·6, 9·4) | | 0·28 | -3·1 (-13·2, 3·1) |
|  | 8 weeks | 24·9 (11·2) | 24·6 (11·8) | 24·3 (7·9) |  | 0·3 | | (-7·1, 7·7) | | | 0·02 |  | -2·7 | | | (-11·2, 5·7) | | -0·27 | -2·4 (-7·1, 7·7) |
|  | 12 weeks | 23·0 (7·1) | 21·9 (8·8) | 22·4 (12·7) | 0·958 | -0·2 | | (-8·2, 7·7) | | | -0·06 | 0·782 | -1·2 | | | (-9·5, 7·2) | | -0·11 | -1·4 (-8·2, 7·7) |
|  | 26 weeks^d^ | 13·2 (11·1) | 18·2 (7·7) | 15·1 (6·4) | 0·849 | -0·9 | | (-10·2, 8·4) | | | -0·04 | 0·256 | 4·6 | | | (-3·3, 12·4) | | 0·48 | -5·5 (-15·8, 4·9) |
| **QIDS Severity** |  | **Rosuvastatin** | **Aspirin** | **Placebo** | **Rosuvastatin and Placebo** | | | | | | | **Aspirin and Placebo** | | | | | | | **Rosuvastatin and Aspirin** |
|  |  | Mean (S_D_) | | | *p*-value | Differential change ^b^ | | 95% CI | | | Effect size^c^ | *p*-value | Differential change ^b^ | | | 95% CI | | Effect size^c^ | Differential change^b^  (95% CI) |
| MADRS  (QIDS<20)  (n=88) | Overall^a^ | - | - | - | 0·694 | - | | - | | | - | 0·673 | - | | | - | | - |  |
|  | Baseline | 31·7 (5·9) | 31·7 (5·1) | 29·5 (5·5) |  |  | | | | | | | | | | | | |  |
|  | 4 weeks | 21·5 (10·4) | 21·5 (9·3) | 22·6 (8·6) |  | -3·2 | | (-8·6, 2·1) | | | -0·3 |  | -3·2 | | | (-7·6, 1·2) | | -0·4 | -3·2 (-8·6, 2·1) |
|  | 8 weeks | 19·2 (11·1) | 19·9 (13·0) | 19·4 (8·9) |  | -2·3 | | (-7·6, 3·1) | | | -0·3 |  | 0·6 | | | (-4·2, 5·5) | | 0·1 | -2·3 (-7·6, 3·1) |
|  | 12 weeks | 17·7 (11·0) | 20·5 (13·8) | 17·7 (11·1) | 0·516 | -2·0 | | (-8·1, 4·1) | | | -0·2 | 0·259 | 3·0 | | | (-2·2, 8·2) | | 0·3 | -2·0 (-8·2, 4·1) |
|  | 26 weeks^d^ | 15·7 (11·8) | 16·3 (10·4) | 11·7 (8·3) | 0·664 | -1·4 | | 9-7·6, 4·8) | | | -0·1 | 0·847 | 0·7 | | | (-6·1, 7·4) | | 0·1 | -0·7 (-7·4, 6·1) |
| MADRS  (QIDS≥20) (n=35) | Overall^a^ | - | - | - | 0·543 | - | | - | | | - | 0·917 | - | | | - | | - |  |
|  | Baseline | 34·4 (5·9) | 37·0 (6·2) | 36·9 (5·2) |  |  | | | | | | | | | | | | |  |
|  | 4 weeks | 26·4 (10·2) | 28·0 (11·5) | 26·5 (10·6) |  | 2·4 | | (-4·2, 8·9) | | | 0·3 |  | 1·7 | | | (-4·7, 8·2) | | 0·3 | 0·7 (-6·1, 7·4) |
|  | 8 weeks | 17·3 (12·8) | 22·5 (13·5) | 23·9 (16·4) |  | -5·6 | | (-13·2, 2·0) | | | -0·5 |  | -4·8 | | | (-14·2, 4·7) | | -0·5 | -3·0 (-13·5, 7·6) |
|  | 12 weeks | 17·1 (12·0) | 22·6 (16·7) | 24·1 (18·0) | **0·045** | -9·5 | | (-18·7, -0·2) | | | -0·8 | 0·378 | -5·2 | | | (-16·7, 6·3) | | -0·3 | -4·2 (-14·5, 6·1) |
|  | 26 weeks^d^ | 18·9 (13·4) | 12·5 (11·6) | 14·0 (17·3) | 0·176 | -7·8 | | (-19·2, 3·5) | | | -0·4 | 0·422 | -4·4 | | | (-15·1, 6·3) | | -0·0 | 4·4 (-6·3, 15·1) |
| **No· MDD EPISODES ≤2, n = 47** |  | **Rosuvastatin** | **Aspirin** | **Placebo** | **Rosuvastatin and Placebo** | | | | | | | **Aspirin and Placebo** | | | | | | | **Rosuvastatin and Aspirin** |
|  |  | Mean (S_D_) | | | *p-*value | Differential change ^b^ | | 95% CI | | | Effect size^c^ | *p*-value | Differential change ^b^ | | | 95% CI | | Effect size^c^ | Differential change^b^  (95% CI) |
| MADRS (no· MDD episodes≤2) (n=47) | Overall^a^ | - | - | - | 0·498 | - | | - | | | - | 0·539 | - | | | - | | - |  |
|  | Baseline | 33·2 (5·5) | 33·0 (5·0) | 31·3 (6·4) |  | | | | | | | | | | | | | |  |
|  | 4 weeks | 23·6 (11·2) | 22·6 (10·0) | 24·5 (9·1) |  | -3·0 | | (-8·9, 2·9) | | | -0·3 |  | -3·8 | | | (-8·9, 1·4) | | -0·5 | 0·9 (-4·6, 6·1) |
|  | 8 weeks | 20·8 (11·0) | 22·5 (11·0) | 21·9 (10·5) |  | -3·6 | | (-9·6, 2·4) | | | -0·4 |  | -1·6 | | | (-7·6, 4·3) | | -0·2 | -2·0 (-7·6, 3·7) |
|  | 12 weeks | 18·7 (11·3) | 22·2 (12·4) | 21·1 (12·7) | 0·151 | -4·6 | | (-10·9, 1·7) | | | -0·5 | 0·848 | -0·6 | | | (-7·0, 5·7) | | -0·1 | -4·0 (-10·3, 2·3) |
|  | 26 weeks^d^ | 19·2 (12·7) | 15·6 (10·5) | 10·9 (7·8) | 0·102 | 5·0 | | (-1·0, 11·0) | | | 0·5 | 0·463 | 2·1 | | | (-3·5, 7·7) | | 0·1 | 2·9 (-3·8, 9·6) |
| MADRS  (no· MDD episodes>2) (n=68) | Overall^a^ | - | - | - |  | - | | - | | | - |  | - | | | - | | - |  |
|  | Baseline | 33·1 (6·2) | 31·5 (6·8) | 33·1 (6·9) |  | | | | | | | | | | | | | |  |
|  | 4 weeks | 21·6 (9·0) | 22·8 (9·5) | 23·0 (9·7) |  | -1·8 | | (-7·1, 3·5) | | | -0·2 |  | 1·2 | | | (-4·5, 6·8) | | 0·1 | -3·0 (-9·1, 3·2) |
|  | 8 weeks | 16·5 (10·4) | 20·9 (11·9) | 22·2 (11·4) |  | -5·5 | | (-11·9, 0·8) | | | -0·6 |  | 0·8 | | | (-5·9, 7·4) | | 0·1 | -6·3 (-13·6, 1·0) |
|  | 12 weeks | 14·4 (8·5) | 24·1 (11·6) | 20·0 (12·7) | **0·045** | -6·6 | | (-13·0, -0·1) | | | -0·7 | 0·147 | 4·9 | | | (-1·7, 11·5) | | 0·5 | -11·5 (-17·7, -5·3) |
|  | 26 weeks^d^ | 11·0 (8·8) | 15·7 (11·1) | 15·3 (12·3) | 0·485 | -2·9 | | (-11·0, 5·2) | | | -0·4 | 0·301 | 5·0 | | | (-4·4, 13·3) | | 0·2 | -7·8 (-17·0, 1·4) |

^a^ Intervention by follow-up interaction test
^b^ Two-way interaction of intervention allocation and measurement time (between group differential change estimated from GEE)
^c^ Cohen’s *d* effect size
^d^ From a GEE that includes baseline, and week 4 to week 26 measures

**Table S5. History and ongoing psychological therapy: comparing Rosuvastatin and Aspirin to placebo**

| Psychological therapy n (% of participants) | | Treatment group | | |
| --- | --- | --- | --- | --- |
|  |  | Rosuvastatin | Placebo | Aspirin |
| History of psychological therapy | Depression | 7 (53.8%) | 3 (27.3%) | 7 (43.8%) |
|  | Depression and anxiety | 3 (23.1%) | 5 (45.5%) | 6 (37.5%) |
|  | Depression and other reasons^*^ | 0 (0.0%) | 0 (0.0%) | 1 (6.3%) |
|  | Other reasons^#^ | 3 (23.1%) | 3 (27.3%) | 2 (12.5%) |
| Ongoing psychological therapy during study | Depression | 33 (66.0%) | 14 (41.2%) | 22 (48.9%) |
|  | Depression and anxiety | 11 (22.0%) | 14 (41.2%) | 13 (28.9%) |
|  | Depression and other reasons^*^ | 1 (2.0%) | 4 (11.8%) | 1 (2.2%) |
|  | Other reasons^#^ | 5 (10.0%) | 2 (5.9%) | 9 (20.0%) |

^*^ Including OCD, Asperger, PTSD, family problems, substance abuse, sexual assault, and bipolar
^#^ Including low mood, social skills building, substance abuse, suicidal ideation, suicidal threats, anger outbursts, employment support, sexual assault, family therapy, eating disorder, and support therapy

**Table S6. History and ongoing antidepressants therapy: comparing Rosuvastatin and Aspirin to placebo**

|  | Antidepressants type | Dosage range (mg) | | Treatment group  n (% of participants) | | | Total |
| --- | --- | --- | --- | --- | --- | --- | --- |
|  |  |  |  | Rosuvastatin | Placebo | Aspirin |  |
| History of antidepressants | Agomelatine^*^ | | 25 | 0 (0.0%) | 1 (2.3%) | 2 (5.6%) | 3 (2.4%) |
|  | Amitriptyline^#^ | | - | 0 (0.0%) | 1 (2.3%) | 0 (0.0%) | 1 (0.8%) |
|  | Cipramil^*^ | | 10 | 0 (0.0%) | 0 (0.0%) | 1 (2.8%) | 1 (0.8%) |
|  | Citalopram | | 20-30 | 1 (2.2%) | 3 (6.8%) | 2 (5.6%) | 6 (4.8%) |
|  | Desvenlafaxine | | 50-150 | 5 (10.9%) | 3 (6.8%) | 2 (5.6%) | 10 (7.9%) |
|  | Duloxetine^#^ | | - | 0 (0.0%) | 1 (2.3%) | 1 (2.8%) | 2 (1.6%) |
|  | Escitalopram | | 5-20 | 10 (21.7%) | 10 (22.7%) | 6 (16.7%) | 26 (20.6%) |
|  | Fluoxetine | | 10-80 | 16 (34.8%) | 14 (31.8%) | 17 (47.2%) | 47 (37.3%) |
|  | Mirtazapine^#^ | | - | 3 (6.5%) | 0 (0.0%) | 1 (2.8%) | 4 (3.2%) |
|  | Paroxetine^#^ | | - | 1 (2.2%) | 1 (2.3%) | 0 (0.0%) | 2 (1.6%) |
|  | Sertraline^#^ | | 50-100 | 6 (13.0%) | 7 (15.9%) | 3 (8.3%) | 16 (12.7%) |
|  | Venlafaxine^*^ | | 275 | 4 (8.7%) | 3 (6.8%) | 1 (2.8%) | 8 (6.3%) |
| Ongoing antidepressants | Citalopram^*^ | | 40 | 1 (7.1%) | 0 (0.0%) | 0 (0.0%) | 1 (2.4%) |
|  | Desvenlafaxine | | 50-100 | 3 (21.4%) | 2 (15.4%) | 3 (20.0%) | 8 (19.0%) |
|  | Duloxetine^#^ | | - | 0 (0.0%) | 1 (7.7%) | 0 (0.0%) | 1 (2.4%) |
|  | Escitalopram | | 10-20 | 2 (14.3%) | 2 (15.4%) | 2 (13.3%) | 6 (14.3%) |
|  | Fluoxetine | | 20-60 | 5 (35.7%) | 4 (30.8%) | 6 (40.0%) | 15 (35.7%) |
|  | Mirtazapine | | 15-30 | 1 (7.1%) | 1 (7.7%) | 1 (6.7%) | 3 (7.1%) |
|  | Paroxetine^#^ | | - | 1 (7.1%) | 0 (0.0%) | 0 (0.0%) | 1 (2.4%) |
|  | Sertraline | | 50-150 | 0 (0.0%) | 2 (15.4%) | 2 (13.3%) | 4 (9.5%) |
|  | Venlafaxine^*^ | | 150 | 1 (7.1%) | 1 (7.7%) | 1 (6.7%) | 3 (7.1%) |

^*^ A single dosage was only reported ^#^ No dosage was reported
